# Influence of diet enriched with conjugated linoleic acids on their distribution in tissues of rats with DMBA induced tumors

**DOI:** 10.1186/1476-511X-9-126

**Published:** 2010-11-02

**Authors:** Agnieszka Białek, Andrzej Tokarz, Agnieszka Dudek, Weronika Kazimierska, Wojciech Bielecki

**Affiliations:** 1 Department of Bromatology, Medical University of Warsaw, Banacha 1, 02-097 Warsaw, Poland; 2 Department of Pathology and Veterinary Diagnostics, Division of Pathomorphology of Animals, Warsaw University of Life Science, Nowoursynowska 159c, 01-776 Warsaw, Poland

## Abstract

**Backround:**

Conjugated linoleic acids (CLA) are a group of positional and geometric isomers of linoleic acid with proven beneficial influence on health. They show e.g. anticarcinogenic, antiobesity, and antiatherogenic effect. Milk, dairy products and meat of poligastric animals are their most valuable dietary sources, with cis-9, trans-11 CLA (RA - rumenic acid) being the predominant isomer. Dietary supplements with CLA became very popular, mainly among the overweight and bodybuilders.

The aim of this study was to examine the influence of the food supplements with conjugated linoleic acid on carcinogenesis in female Sprague-Dawley rats and evaluation of CLA and other fatty acids distribution in their bodies.

Animals were divided into four groups depending on the diet supplementation (oil or Bio-C.L.A. (Pharma Nord Denmark) given intragastrically) and presence or absence of carcinogenic agent (7,12-dimethylbenz[a]antharcene). Animals were decapitated at 21st week of experiment and serum and microsomes were extracted.

**Results and conclusions:**

The mammary tumours (adenocarcinoma) occurred in groups treated with DMBA. Diet enriched with CLA decreased the cancer morbidity (67% in Bio-C.L.A. compared to 88% in oil) and delayed the cancer induction (p = 0.0018). There were no differences in body and organs weight.

The supplement used in the study was a mixture of several fatty acids with the greatest proportion of CLA isomers: trans-10, cis-12 (33%) and cis-9, trans-11 (31%). Both of them were present in tissues but the content of rumenic acid was greater. Dietary supplementation had also significant impact on other fatty acids content, both in serum and in microsomes.

## Background

Conjugated Linoleic Acid (CLA) is a term for positional and geometrical isomers of octadecadienoic acid with two double bonds separated by only one single bond. They are found in various types of food, mainly in milk and dairy products and meat of ruminants. The predominant CLA isomer in food is *cis*-9, *trans*-11-octadecadienoate (Rumenic Acid - RA), which constitutes over 90% of all CLA isomers [1]. Their content in food differs and depends on many factors such as animal species, season, way of feeding, place of pasture [2-6]. Commercially available supplements are the mixture of two main isomers: cis-9, trans-11 CLA and trans-10, cis-12 CLA in equal proportions. This group of fatty acids has been extensively studied for recent years, in both in vivo and in vitro models, because of their beneficial biological effects: protection against cancer [7-10], prevention of atherosclerosis [11-14], reduction of obesity [15-17] and hypertension [18]. However, despite numerous experiments their mechanism of action is still under investigation.

As it is known that many cancers are associated with diet, especially with dietary fat, we have focused on changes in fatty acids profile as a possible effect of CLA supplementation. The objectives of the present study were: to monitor the influence of CLA supplementation on mammary carcinogenesis in rats, to compare the CLA distribution in tissues and to assess their influence on other fatty acids profile.

## Materials and methods

### Animals

**Figure 1:**
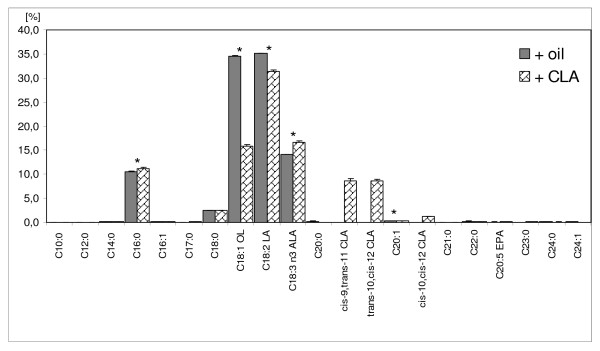
**Fatty acids composition of experimental diets (% of total fatty acids)**.

This research and guiding principles in the care of laboratory animals were approved by The Local Ethic Commission on Animal Experiments. Female Sprague-Dawley rats (n = 33) were purchased at 30 day of age from Division of Experimental Animals, Department of General and Experimental Pathology (Medical University of Warsaw, Warsaw, Poland).The animal room was kept at 21°C, in a 12 h light : 12 h dark cycle. A standard diet composed of 22.0% protein, 4.0% fat, 30.0% starch, 5.0% fibre, 6.5% minerals (Labofeed H, Wytwórnia pasz "Morawski", Żurawia 19, Kcynia, Poland) was fed ad libitum. The daily food consumption was about 10 g per rat. After 1-week adaptation animals were randomly divided into four groups of 8 - 9 each. All rats from groups A1 and B1 received intragastrically at 50 days of age a single dose of 80 mg/kg body weight of carcinogenic agent - DMBA (7,12-Dimethylbenz[a]anthracene, approx. 95%, Sigma-Aldrich) for the induction of mammary tumours. From that day all animals were fed different dietary supplementation (oil - groups A1 and G1, or Bio-C.L.A. (Pharma Nord Denmark) - groups B1 and D1, given intragastrically 0.15 cm^3^/day) for the following 15 weeks. During this period rats were palpated weekly to detect the appearance of tumours. Table 1 shows the characteristic of experimental groups. The fatty acids daily intake is shown in Figure 1. The experiment was terminated at the end of 21 week. All animals were decapitated and exsanguinated.

**Table 1:** Characteristics of experimental groups

Group	Number	Supplementation	Carcinogenic agent	Morbidity	Age of tumour appearance (day of life) mean ± SD	Number of tumours per individual mean ± SD	Tumour weight (g) mean ± SD
A1	8	+ oil	+ DMBA	88%	106 ± 14*	1,6 ± 1,1	3,76 ± 3,37
B1	9	+ CLA	+ DMBA	67%	143 ± 19*	0,9 ± 0,8	2,69 ± 2,32
G1	8	+ oil	-	-			
D1	8	+ CLA	-	-			

(* significant differences between groups in Student's t - test)

### Histopathological examination

The effectiveness of cancer induction in DMBA-treated groups (A1 and B1) was determined as the percentage of animals with tumours. Tumours were collected during necropsy and some of them were fixed in 10% formalin. They were identified as adenocarcinomas and papillary adenocarcinomas of mammary gland. In non-DMBA-treated groups (D1 and G1) there were no spontaneous tumours.

### Preparation of serum and microsomes

Serum was obtained by the centrifugation of blood for 10 min at 3000 rpm at 4°C and stored at -20°C in Eppendorf test tubes until further analysis.

Hepatic microsomes were prepared according to modification of the Kłyszejko-Stefanowicz method [19]. A sample of liver (4 g) was mechanically homogenized in saccharose solution (16 cm^3^; 0.25 mol/dm3) buffered with TRISS-buffer (pH 7.4). Homogenate was centrifuged for 10 min at 4000 rpm at 4°C. The sediment was discarded and the supernatant fluid was centrifuged for 20 min at 16 000 × g at 4°C. The sediment was discarded and the supernatant was centrifuged for 75 min at 100 000 × g at 4°C. The supernatant was discarded and the pellet was resuspended in 4 cm^3 ^0.25 mol/dm3 saccharose solution. The suspension of hepatic microsomes was stored in Eppendorf test tubes at -20°C until further analysis.

### Fatty acids analysis

Fatty acid analysis was made with GC with capillary column and flame-ionization detection.

The rat serums were thawed only once and samples of 100 μl were trans-esterificated according to the procedure of Bondia-Pons et al. with slight modifications [20]. Without prior lipids extraction each sample was hydrolyzed by heating with 2.5 cm^3 ^sodium methylate reagent (0.5 mol/dm^3^) at 80°C and fatty acids were converted to methyl esters by heating with 2.5 cm^3 ^of 14% boron trifluoride-methanol reagent at 80°C for 3 min. FAME were isolated with hexane (2 × 0.5 cm^3^) after adding 1.0 cm3 of saturated sodium chloride solution. Organic extracts were dried with anhydrous sodium sulphate and evaporated to dryness under a stream of nitrogen. FAME were diluted in 20 μl of hexane and stored at -20°C. They were separated and quantified by GC using Shimadzu GC-17A chromatograph equipped with flame ionization detector. Injector was heated to 250°C and detector was heated to 270°C. Separation was performed on BPX 70 capillary column (60 m × 0.25 mm i.d., film thickness: 0.20 μm, SGE) with helium as the carrier gas. The initial oven temperature was 140°C for 1 min, thereafter increased by 20°C/min to 200°C and held for 20 min and then increased by 5°C/min to 220°C and held for 25 min. The whole analysis lasted 53 min. FAME standards (Supelco 37 Component FAME Mix), CLA FAME reference standard (Nu-Chek-Prep) and RA FAME standard (methyl 9 cis 11 trans conjugated linoleate, Nu-Chek-Prep) were used to identify the fatty acids present in samples.

The hepatic microsomes samples were thawed only once and lipids were extracted according to Folch et al. with slight modification [21]. 200 μl of microsomal suspensions were mixed with 2.5 cm^3 ^chloroform : methanol (2:1, v/v) and after vigorous shaking the chloroform layer was separated. The residue was mixed with 1.5 cm^3 ^chloroform : methanol (2:1, v/v) and the extraction was repeated. Combined chloroform layers were centrifuged for 10 min at 3000 rpm and the sediment was discarded. The organic extract was evaporated to dryness under a stream of nitrogen. The residue was taken for the preparation of FAME according to procedure previously described for serum.

The protein content of hepatic microsomes was measured by the method of Lowry et al. [22].

### Statistical analysis

Results were evaluated with Statistica 9.0 (StatSoft, Poland). The data were tested for normality with Shapiro - Wilk test. The Student's t-test and ANOVA were used to test differences between groups for variables following normality and Mann - Whitney U-test and Kruskal - Wallis test was performed in case of the shortage of normal distribution. P - value ≤ 0.05 was considered significant. All data are mean values ± standard deviation.

## Results and discussion

The percentage of tumour-bearing animals in each DMBA-treated group is shown in Table 1. The higher effectiveness of cancer induction was observed in A1 group supplemented with oil. 7 of 8 animals in this group developed tumours, and 2 of them had 3 tumours, 2 had 2 tumours and 3 had 1 tumour. CLA supplementation decreased the incidence of tumours (67% in B1 group in comparison with 88% in A1 group). 6 of 9 rats supplemented with CLA developed tumours, and 2 animals had 2 tumours each. There were no significant differences in the mean number of tumours per rat in DMBA-treated groups although the percentage of animals with multiple tumours was much higher in group supplemented with oil than in CLA supplemented group (57% in A1 group in comparison with 33% in B1 group). That confirms the greater efficiency of DMBA-induced mammary carcinogenesis in A1 group and suggests the greater invasiveness of those tumours. There were no significant differences in the mean weight of single tumour between DMBA-treated groups but we observed the tendency to developing heavier tumours in A1 group supplemented with oil. However 2 of 3 single tumours appeared to be much heavier than the rest of tumours in A1 group (10.50 g and 11.93 g respectively) and the range of tumour weight was wider in A1 group in comparison with B1 group. In B1 group there was only 1 single tumour much heavier than the rest of them (8.51 g). Moreover the first tumour in group A1 appeared on average 5 weeks earlier than in group B1 (p = 0.0018). That indicates the ability of CLA to inhibit the mammary adenocarcinoma development. The lack of significant differences in the tumours weight and in the number of tumours per rat between A1 and B1 groups may be the result of the strength of action of applied carcinogenic agent in the experimental conditions. 7,12-Dimethylbenz[a]anthracene administered intragastrically in a single dose of 80 mg/kg body weight appeared to be so effective in the induction of mammary tumours in both groups, that the preventive action of Conjugated Linoleic Acid was not efficient enough and confirmed with significant differences in all examined parameters. Observed retardation and the tendency to develop fewer and slighter tumours in CLA supplemented group clearly indicates the protective meaning of this compounds in mammary carcinogenesis.

FAME profiles of serum and microsomes were measured using GC. In our experiment we analysed 21 fatty acids. C20:4 n-6, C18:2 n-6, C16:0, C18:0 and C18:1 n-9 were found to be the main fatty acids in the serum of all investigated groups (Table 2) whereas C18:0, C20:4 n-6, C16:0, C18:2 n-6 and C22:6 n-3 were the most common in the hepatic microsomes (Table 3). Our observations were similar to those of other authors. Bondia-Pons et. al found C18:2 n-6, C16:0, C20:4 n-6, C18:1 n-9 and C18:0 to be the most prominent in rats' plasma [20]. The slight differences e.g. in share of AA could have been caused by different fatty acids profiles of applied diets. There were significant differences in fatty acid concentration among examined groups. A1 showed the highest concentration of EPA in serum and microsomes, significantly higher than other groups. Also C15:0 amount in microsomes of A1 was considerably greater. In contrast, the C12:0 content in serum samples of this group was the lowest. We observed significantly higher concentration of C12:0 and C17:1, and significantly lower concentration of C18:3 n-3 and C22:6 n-3 in serum of G1. The highest C18:1 n-9 level was observed in the samples of serum obtained from rats in groups A1 and G1, whose diet contained noticeably more of this fatty acid (Figure 1). Similar dependence was evident for the C22:6 n-3 concentration. Jelińska et al. found that higher concentration of C18:3 n-3 in administered diet increased the EPA and DHA levels in liver phospholipids of both non-DMBA- and DMBA-treated rats [23]. Diet of B1 and D1 group, supplemented with Bio-C.L.A., was significantly richer in C18:3 n-3 which caused the increased content of this fatty acid's metabolite - C22:6 n-3 in serum of these groups. DHA concentration in hepatic microsomes was also remarkably higher in CLA-treated groups. This observation is in agreement with that of Eder et al. [24], who measured higher concentration of C22:6 n-3 and total n-3 PUFA in liver phosphatidylcholine of rats fed CLA-enriched diet. Moreover, they detected in this group the increased gene expression of Δ6-desaturase and increased conversion of C18:3 n-3, but they linked that with the low concentration of C18:2 n-6 in that diet. However, in our study the LA was the predominant fatty acid in B1 and D1 group (Figure 1). That supports the hypothesis about the dependence between CLA supplementation and ALA metabolism. Senkal et al. observed that administration of long chain n-3 PUFA enriched diets lead to increased incorporation of EPA and DHA both in serum and tissues (liver, gut mucosa and tumour). They claim that diet can modify not only lipid profile of different tissues but also their immune response, especially inflammatory response [25]. The data presented by Eder et al. show that increased CLA intake and decreased n-6 fatty acids administration can lower the concentration of several eicosanoids, which indicates that CLA can also modulate the immune response [24]. However Banni at al., who used in their experiment CLA-enriched butter, observed the differences in the concentration of C18:1, C18:2, C18:3 between the two dietary groups, in both neutral lipids and phospholipids. Only C20:4 content in some of the phospholipid fractions was significantly lower in group with high CLA intake [26]. That disagrees with our results.

**Table 2:** Fatty acids profile in serum of investigated dietary groups

	A1	B1	G1	D1	
	
	*Mean *± SD [%]				p value < 0,05
C12:0	0,03 ± 0,01	0,04 ± 0,01	0,05 ± 0,01	0,04 ± 0,01	0,0234
C14:0	0,25 ± 0,04	0,24 ± 0,03	0,25 ± 0,05	0,25 ± 0,02	
C15:0	0,36 ± 0,04	0,35 ± 0,06	0,27 ± 0,06	0,32 ± 0,05	0,0135
C16:0	17,87 ± 1,83	17,75 ± 1,36	16,21 ± 1,33	16,50 ± 1,11	
C16:1	0,96 ± 0,57	0,52 ± 0,13	0,56 ± 0,18	0,50 ± 0,11	
C17:0	0,51 ± 0,06	0,51 ± 0,08	0,53 ± 0,08	0,55 ± 0,05	
C17:1	0,07 ± 0,03	0,06 ± 0,01	0,09 ± 0,01	0,08 ± 0,01	0,0121
C18:0	13,71 ± 1,61	14,38 ± 1,25	16,98 ± 0,67	15,98 ± 1,11	0,0000
C18:1 *n*-9	9,14 ± 1,38	7,65 ± 0,64	8,56 ± 0,89	7,19 ± 1,01	0,0075*
C18:2 *n*-6	21,16 ± 1,25	21,81 ± 0,72	18,49 ± 1,29	20,04 ± 1,03	0,0000
C18:3 *n*-6	0,36 ± 0,07	0,29 ± 0,05	0,39 ± 0,05	0,39 ± 0,06	0,0050
C18:3 *n*-3	2,52 ± 0,48	2,86 ± 0,37	2,14 ± 0,41	2,34 ± 0,39	0,0085
C20:0	0,10 ± 0,04	-	0,07 ± 0,01	0,06	
*cis*-9, *trans*-11 CLA	0,05 ± 0,03	0,40 ± 0,12	-	0,34 ± 0,11	0,0037
*trans*-10, *cis*-12 CLA	-	0,16 ± 0,08	-	0,10 ± 0,04	
C20:3 *n*-6	0,66 ± 0,28	0,42 ± 0,06	0,36 ± 0,05	0,36 ± 0,05	0,0026*
C20:4 *n*-6	18,93 ± 3,88	20,05 ± 1,03	24,20 ± 2,01	23,44 ± 1,85	0,0004*
C20:3 *n*-3	0,21 ± 0,08	0,18 ± 0,10	0,21 ± 0,03	0,22 ± 0,10	
C20:5 *n*-3	2,70 ± 0,40	2,08 ± 0,41	1,65 ± 0,32	2,01 ± 0,30	0,0000
C24:0	0,09 ± 0,03	0,09 ± 0,01	0,07 ± 0,02	0,06 ± 0,01	0,0315
C22:6 *n*-3	4,13 ± 0,50	4,44 ± 0,71	3,75 ± 0,37	4,45 ± 0,38	0,0315

(p value < 0,05 for those fatty acids with significant differences among groups in ANOVA or * Kruskal - Wallis test).

**Table 3:** Fatty acids profile in hepatic microsomes of investigated dietary groups

	A1	B1	G1	D1	
	
	*Mean *± SD [%]				p value < 0,05
C12:0	0,08 ± 0,05	0,05 ± 0,02	0,05 ± 0,02	0,07 ± 0,03	
C14:0	0,29 ± 0,10	0,22 ± 0,05	0,22 ± 0,08	0,22 ± 0,06	
C15:0	0,25 ± 0,04	0,22 ± 0,03	0,20 ± 0,06	0,20 ± 0,03	0,0065
C16:0	18,38 ± 1,54	18,63 ± 1,76	16,09 ± 1,31	16,62 ± 1,01	0,0019
C16:1	0,57 ± 0,32	0,32 ± 0,10	0,36 ± 0,19	0,23 ± 0,05	0,0140*
C17:0	0,64 ± 0,09	0,62 ± 0,09	0,64 ± 0,10	0,67 ± 0,06	
C17:1	0,07 ± 0,02	0,07 ± 0,01	0,11 ± 0,02	0,10 ± 0,02	0,0001
C18:0	23,48 ± 3,13	23,33 ± 1,72	25,94 ± 1,95	27,32 ± 2,01	0,0023
C18:1 *n*-9	8,03 ± 3,88	5,80 ± 0,68	6,67 ± 1,47	4,59 ± 0,76	0,0009*
C18:2 *n*-6	14,37 ± 1,59	15,47 ± 1,11	13,84 ± 1,19	13,56 ± 1,61	0,0383
C18:3 *n*-6	0,18 ± 0,04	0,15 ± 0,04	0,21 ± 0,03	0,20 ± 0,04	0,0061
C18:3 *n*-3	1,53 ± 0,30	1,86 ± 0,32	1,39 ± 0,32	1,29 ± 0,39	0,0068
C20:0	0,08 ± 0,03	0,05	0,07 ± 0,06	0,09	
*cis*-9, *trans*-11 CLA	0,09 ± 0,06	0,33 ± 0,09	-	0,25 ± 0,10	0,0128
*trans*-10, *cis*-12 CLA	-	0,16 ± 0,08	-	0,08 ± 0,05	
C20:3 *n*-6	0,69 ± 0,29	0,44 ± 0,07	0,40 ± 0,10	0,41 ± 0,07	0,0022*
C20:4 *n*-6	16,67 ± 2,79	17,28 ± 0,64	20,31 ± 1,79	19,82 ± 1,20	0,0003
C20:3 *n*-3	0,16 ± 0,05	0,14 ± 0,06	0,12 ± 0,02	0,11 ± 0,07	
C20:5 *n*-3	1,29 ± 0,26	0,99 ± 0,23	0,84 ± 0,15	0,99 ± 0,12	0,0011
C24:0	0,10 ± 0,04	0,08 ± 0,02	0,06 ± 0,02	0,05 ± 0,01	0,0071*
C22:6 *n*-3	7,14 ± 0,92	8,36 ± 1,08	7,41 ± 0,99	8,30 ± 0,98	0,0375

(p value < 0,05 for those fatty acids with significant differences among groups in ANOVA or * Kruskal - Wallis test).

Moreover, not only diet but also carcinogenic agent treatment had great influence on fatty acids profile, both in serum and in microsomes of the investigated groups. The lover levels of C17:1, C18:0, C18:2 n-6, C20:4 n-6 were observed in serum and microsomes of DMBA-treated groups, whereas C18:2 n-6 and C18:3 n-3 showed the opposite tendency. Hoffmann et al. also observed differences between fatty acids profile of healthy and cancerous tissues, especially the n-3 and n-6 fatty acids content [27,28].

Two main CLA isomers were detected in all samples of serum and microsomes obtained from CLA supplemented groups and only in few samples from A1 group. The CLA isomers were separated into two peaks, of which the first one was identified as the cis-9, trans-11 CLA and the second one as the trans-10, cis-12 CLA. Bio-C.L.A. used as the main source of CLA for B1 and D1 group consisted of several fatty acids, with prevailing share of two CLA isomers: cis-9, trans-11CLA: 33% and cis-9, trans-11 CLA: 31%. However RA was shown to be the prominent CLA isomer in each study group. Its mean content in serum was 0.37 ± 0.11% of total fatty acids whereas trans-10, cis-12 CLA constituted only 0.13 ± 0.07% of them. There were no significant differences in RA content in serum of B1 and D1 groups and its concentration in A1 was significantly lower than in CLA supplemented groups (Table 2). Zlatanos et al. in their experiment gave volunteers similar CLA supplement with two major isomers: cis-9, trans-11 and trans-10, cis-12 CLA (1:1), and also detected only RA in the amount of 0.20 ± 0.02% whereas the other isomer was below the detection limit [29]. Burdge et al. got similar results for the plasma phosphatidylcholine, where cis-9, trans-11 CLA was the predominant isomer, even when the trans-10, cis-12 CLA was the main ingredient of the administrated dietary supplement [30]. The mean content of RA in hepatic microsomes was 0.27 ± 0.11% of total fatty acids, and the concentration of the second identified CLA isomer was 0.12 ± 0.07%. We observed similar lack of differences in RA amount in microsomes between B1 and D1, and the significant lower content of this fatty acid in A1 (Table 3) as in serum samples.

**Figure 2:**
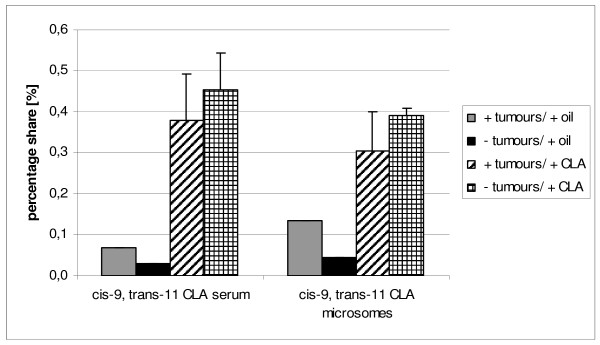
**Content of cis-9, trans-11 CLA in serum and microsomes of individuals with or without tumours**.

**Figure 3:**
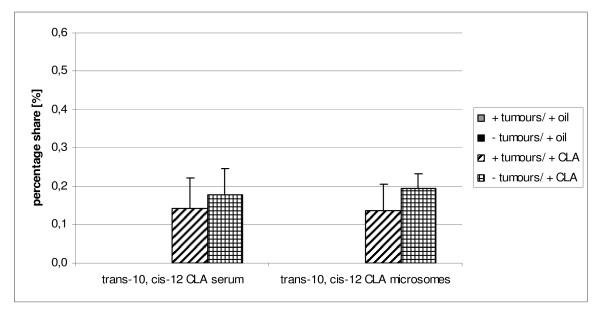
**Content of trans-10, cis-12 CLA in serum and microsomes of individuals with or without tumours**.

We compared the CLA isomers concentration in serum and microsomes within A1 and B1 groups between tumour-bearing and non-tumour-bearing animals (Figure 2 and Figure 3). There were no significant differences either in cis-9, trans-11 CLA nor trans-10, cis-12 CLA content among the groups although the CLA amount seemed to be slightly higher in serum of B1 rats without tumours in comparison with B1 tumour-bearing animals (0.45 ± 0.09% versus 0.38 ± 0.12% for cis-9, trans-11 CLA, and 0.18 ± 0.07% versus 0.14 ± 0.08% for trans-10, cis-12 CLA). Comparison of CLA isomers distribution in hepatic microsomes of B1 revealed the great similarity to their distribution in serum (0.39 ± 0.02% versus 0.31 ± 0.10% for cis-9, trans-11 CLA, and 0.20 ± 0.04% versus 0.14 ± 0.07% for trans-10, cis-12 CLA). This observation is in disagreement with the results of Hoffmann et al., who found CLA content to be significantly higher in total cancerous testicular tissue than in normal. Only in mitochondrial fraction of cancerous tissue CLA concentration tended to be lower than in normal tissue, whereas other subcellular fractions showed the opposite tendency [28].

**Figure 4:**
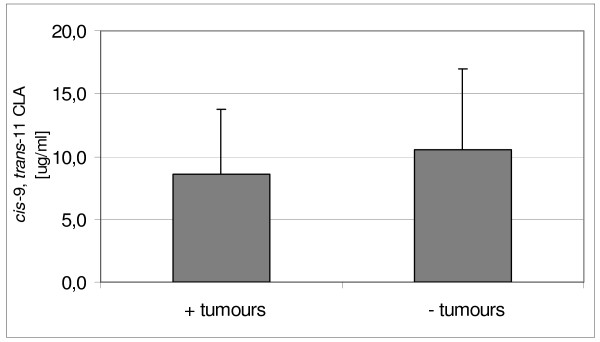
**Content of cis-9, trans-11 CLA in serum of B1 individuals with or without tumours**.

**Figure 5:**
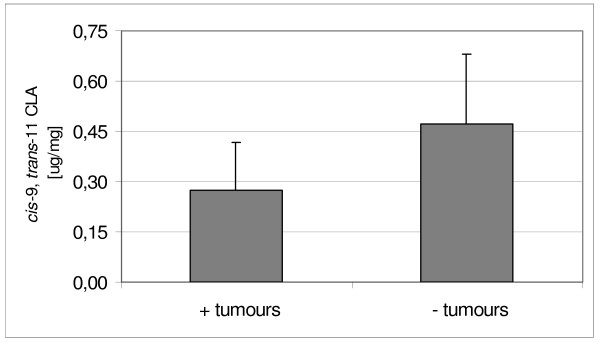
**Content of cis-9, trans-11 CLA in microsomes of  B1 individuals with or without tumours**.

Concentration of dominant RA in serum of rats fed CLA diets were 9.25 ± 5.28 μg/cm3 (B1) and 6.27 ± 4.05 μg/cm3 (D1) respectively and they did not differ significantly. Figure 4 shows the comparison of absolute RA content in B1 serum between individuals with or without developed tumours. The cis-9, trans-11 CLA content in samples obtained from individuals without noticeable tumours tended to be higher than in tumour-bearing (10.51 ± 6.48 μg/cm3 versus 8.63 ± 5.13 μg/cm3), although no significant differences were detected. The total concentration of RA in hepatic microsomes suspension was calculated to the protein content. Its content in CLA supplemented groups was similar (B1: 0.34 ± 0.18 μg/mg, and D1: 0.26 ± 0.29 μg/mg) and the distribution pattern of this isomer in B1, depending on tumour development, was very similar to its distribution in serum (Figure 5). We observed a slight increase in RA content in non-tumour-bearing individuals (0.47 ± 0.41 μg/mg versus 0.28 ± 0.14 μg/mg).

## Conclusions

Our findings confirm the great significance of Conjugated Linoleic Acid content in the diet on mammary carcinogenesis. CLA can inhibit the mammary tumours development and also influence the other fatty acids profile in tissues. Its content seemed to be slightly higher in serum and microsomes of rats without tumours in comparison with tumour-bearing animals. They also show that both type and amount of fatty acids in diet and environmental factors, such as carcinogenic agents, influence the fatty acids profile. On the other hand changes in fatty acids profile caused by CLA supplementation can explain the numerous activities of these fatty acids.

## Abbreviations

CLA: Conjugated linoleic acid; RA: Rumenic acid; FAME: Fatty acid methyl ester; DMBA: 7,12-dimethylbenz[a]anthracene; GC: gas chromatography; DHA: Docosahexaenoic acid; EPA: Eicosapentaenoic acid; AA: Arachidonic acid; LA: Linoleic acid; ALA: α-linolenic acid

## Competing interests

The authors declare that they have no competing interests.

## Authors' contributions

AB conceived, designed and carried out the experiment and performed the statistical analysis. AT coordinated the study. AD and WK carried out the GC analysis. WB carried out the histopathological examination. All authors read and approved the final manuscript.
